# Effect of the perception of breakfast consumption on subsequent appetite and energy intake in healthy males

**DOI:** 10.1007/s00394-021-02727-5

**Published:** 2021-11-11

**Authors:** Tommy Slater, William J. A. Mode, John Hough, Ruth M. James, Craig Sale, Lewis J. James, David J. Clayton

**Affiliations:** 1 Musculoskeletal Physiology Research Group, Nutrition and Exercise Physiology, Sport, Health and Performance Enhancement Research Centre, School of Science and Technology, Clifton Campus, Nottingham Trent University, Nottingham, NG11 8NS Nottinghamshire UK; 2 National Centre for Sport and Exercise Medicine, School of Sport, Exercise and Health Sciences, Loughborough University, Loughborough, LE11 3TU Leicestershire UK

**Keywords:** Breakfast skipping, Energy intake, Energy balance, Appetite hormones, Placebo feeding

## Abstract

**Purpose:**

This study aimed to assess the effects of consuming a very-low-energy placebo breakfast on subsequent appetite and lunch energy intake.

**Methods:**

Fourteen healthy males consumed water-only (WAT), very-low-energy, viscous placebo (containing water, low-calorie flavoured squash, and xanthan gum; ~ 16 kcal; PLA), and whole-food (~ 573 kcal; FOOD) breakfasts in a randomised order. Subjects were blinded to the energy content of PLA and specific study aims. Venous blood samples were collected pre-breakfast, 60- and 180-min post-breakfast to assess plasma acylated ghrelin and peptide tyrosine tyrosine concentrations. Subjective appetite was measured regularly, and energy intake was assessed at an ad libitum lunch meal 195-min post-breakfast.

**Results:**

Lunch energy intake was lower during FOOD compared to WAT (*P* < 0.05), with no further differences between trials (*P* ≥ 0.132). Cumulative energy intake (breakfast plus lunch) was lower during PLA (1078 ± 274 kcal) and WAT (1093 ± 249 kcal), compared to FOOD (1554 ± 301 kcal; *P* < 0.001). Total area under the curve (AUC) for hunger, desire to eat and prospective food consumption were lower, and fullness was greater during PLA and FOOD compared to WAT (*P* < 0.05). AUC for hunger was lower during FOOD compared to PLA (*P* < 0.05). During FOOD, acylated ghrelin was suppressed compared to PLA and WAT at 60 min (*P* < 0.05), with no other hormonal differences between trials (*P* ≥ 0.071).

**Conclusion:**

Consuming a very-low-energy placebo breakfast does not alter energy intake at lunch but may reduce cumulative energy intake across breakfast and lunch and attenuate elevations in subjective appetite associated with breakfast omission.

**Trial registration:**

NCT04735783, 2nd February 2021, retrospectively registered.

**Supplementary Information:**

The online version contains supplementary material available at 10.1007/s00394-021-02727-5.

## Introduction

Obesity is a risk factor for several chronic diseases including type-2 diabetes, heart disease and some forms of cancer [1]. Recent predictions estimate that even in the best case scenario, the majority of the English population will be at increased risk of disease because of excess body weight until at least the year 2035 [2]. It has been suggested that action taken to prevent weight gain will yield greater success than action taken to treat obesity due to the energy balance system showing a stronger opposition to weight loss than weight gain [3]. Therefore, it is important that preventative action is taken by lean individuals, who may yet develop overweight or obesity later in life. Obesity is caused by a sustained positive energy imbalance, in which energy intake exceeds energy expenditure [3, 4], although the underlying causes of this positive energy imbalance are wide-ranging and complex. Reducing daily energy intake is a seemingly simple solution to this, although numerous factors often impede the long-term success of such interventions, including the potential for compensatory alterations in appetite regulation that stimulate an increase in energy intake [5].

Extending the naturally occurring overnight fasting period, thereby restricting the time available for food intake, has emerged as a simple and effective dietary strategy for reducing daily energy intake that may assist with weight management [6–8]. Randomised, control trials have been utilised to isolate and examine the effects of breakfast omission on energy balance by either providing or withholding breakfast. In an acute, laboratory-controlled setting, a single omission of breakfast typically elicits elevations in subjective appetite during the morning [9–12], elevated concentrations of the appetite-stimulating hormone ghrelin [13], and reductions in appetite suppressing hormones such as peptide tyrosine tyrosine (PYY) [9, 12, 14]. These appetite responses to breakfast omission often lead to increased energy intake at lunch [10, 12, 15, 16], although the absolute increase in energy intake at this meal is rarely large enough to fully compensate for energy omitted at breakfast. As such, breakfast omission typically reduces daily energy intake [10, 12, 16], although this is not a universal finding, as one study observed complete energy intake compensation at lunch [15]. Furthermore, longer-term studies have observed increased self-reported daily energy intake during 2 weeks of breakfast omission in lean females [17], and that increased energy intake over the day completely compensated for the energy omitted at breakfast during 6 weeks breakfast omission in individuals living with obesity [18]. These studies highlight the challenge of compensatory eating associated with chronic breakfast omission. It is, therefore, important to explore potential strategies that can attenuate elevations in appetite and subsequent energy intake in response to breakfast omission.

The inability to blind participants to breakfast omission causes a problem for the interpretation of data from these studies, as the participants are acutely aware of whether or not they have consumed breakfast [19]. Placebo-controlled study designs are used in research to dissociate the physiological and psychological effects of an intervention and have been recently employed in the context of breakfast omission. Consuming a virtually energy-free ‘placebo’ breakfast has been shown to suppress subjective appetite compared to plain water, and by a similar extent to an energy-containing (~ 496 kcal) breakfast meal [20]. Somewhat contrastingly, the high energy-containing breakfast suppressed total plasma ghrelin concentrations, whereas the placebo breakfast had no effect on plasma ghrelin concentrations, mirroring the response to the plain water trial. This disparity between the subjective and physiological markers of appetite indicates that the response to breakfast may, at least partly, be due to psychological factors associated with the act of consuming breakfast, rather than physiological effects related to nutrient consumption per se. Importantly, eating behaviour does not universally correspond to changes in subjective appetite or concentrations of ghrelin and PYY [9, 21, 22]; therefore, whether the observed suppression of subjective appetite sensations following placebo breakfast consumption manifests in a reduction in energy intake at a subsequent eating occasion is not known.

The aims of this study were to examine the effects of a very-low-energy, viscous placebo breakfast on subjective appetite, peripheral appetite-regulatory hormone concentrations, and subsequent energy intake at an ad libitum lunch, compared to a typical whole-food breakfast and a water-only control. We hypothesised that the whole-food and placebo breakfast meals would similarly suppress subjective appetite sensations compared to the water-only control, and that these changes would result in comparable reductions in energy intake at lunch.

## Methods

### Subjects

This study was conducted according to the guidelines laid down in the 1964 Declaration of Helsinki and its later amendments, and all procedures were approved by the Nottingham Trent University Ethical Advisory Committee; ethics application number: 632. All subjects completed a health screening questionnaire and provided written informed consent before commencing the study. Fourteen healthy, weight-stable (self-reported), males completed the study (Table 1). For enrolment onto the study, subjects were required to be non-smokers who regularly consumed breakfast and did not exhibit restrained, disinhibited, or hungry eating tendencies [23]. Given the lack of published data to inform a sample size calculation, the sample size used was similar to previous studies from our group which assessed energy intake at lunch in response to breakfast omission [10, 11].

**Table 1:** Participant baseline characteristics (*n* = 14)

Characteristic	Mean	SD
Age (years)	24	2
Weight (kg)	77.1	6.8
Height (m)	1.81	0.07
BMI (kg/m^2^)	23.5	2.3
Body fat (%)	13.2	3.4
Dietary restraint^a^	6	2
Dietary disinhibition^a^	5	2
Hunger^a^	6	3
Estimated resting metabolic rate (kcal/day)^b^	1792	93

^a^Three-factor eating questionnaire[23]

^b^Estimated via predictive equation[26]

### Study design

Subjects completed a preliminary trial, followed by three experimental trials which were completed in randomised, cross-over order (randomisation by drawing trial orders for subjects out of a bag containing the six possible combinations). Each experimental trial involved the consumption of a different breakfast, before energy intake was assessed at an ad libitum lunch meal 195 min later. The breakfasts investigated were a very-low-energy, viscous placebo breakfast (PLA), a typical whole-food breakfast (FOOD), and a water-only control (WAT). Subjects were not told that the PLA breakfast contained almost no energy or the aims or hypotheses of the study. They were informed that the purpose was to compare subjective and physiological responses to a ‘novel breakfast’. Following completion of the final experimental trial, the contents of the PLA breakfast were revealed to the subjects, and they were informed of the true aims of the study.

### Preliminary trial

Subjects’ body mass (to the nearest 0.1 kg; Adam CFW150; Adam Equipment Limited; Milton Keynes; UK) and height (to the nearest 0.01 m; Seca; Hamburg; Germany) were measured, before skinfold callipers were used at four upper-body sites (biceps, triceps, subscapular and iliac crest; Harpenden, West Sussex, UK) to estimate body fat percentage [24]. Subjects were also familiarised to the ad libitum lunch meal procedures used in experimental trials (explained in detail below).

### Pre-trial standardisation

In the 24 h prior to the first experimental trial, subjects recorded all dietary intake and physical activity. This was then replicated during the 24 h preceding the remaining two experimental trials. Subjects were strictly instructed to refrain from strenuous physical activity and alcohol intake in the 24 h before each experimental trial. Subjects’ adherence with these standardisation measures were confirmed verbally before each experimental trial. All experimental trials commenced at the same time of day and were separated by at least 4 days.

### Protocol

Subjects arrived at the laboratory at 08:30 following a ≥ 11 h overnight fast (water was permitted overnight, but volume was standardised) and rested in a supine position for 20 min before baseline venous and capillary blood samples were collected. Baseline measures of subjective appetite were obtained using a visual analogue scale immediately before subjects were provided with their allocated breakfast meal (0 min), which was consumed in its entirety within 10 min. A second subjective appetite measurement was obtained immediately after breakfast consumption (10 min). Subjects then rested quietly in the laboratory, with subjective appetite measurements collected at 30, 60, 120, and 195 min; capillary blood samples were collected at 30, 60, 90, 120, and 180 min; and venous blood samples were collected after 20 min of supine rest at 60 and 180 min. Subjects did not consume any additional water between breakfast and lunch. An ad libitum pasta lunch meal was served at 195 min, and subjects were permitted 20 min to eat. Subjective appetite measurements were obtained immediately before, and immediately (215 min) and 60 min (275 min) after the eating period. Subjects did not consume any additional food or fluids until after the final appetite measurement was completed.

### Breakfast meals

During PLA, subjects consumed a viscous breakfast meal with a volume equating to 5 mL/kg body mass. The meal consisted of 15% (0.75 mL/kg body mass) low-energy flavoured squash (Vimto—No Added Sugar Squash, Vimto, Warrington, UK), with the remainder made up of tap water. To thicken the solution and increase the perception of energy intake [25], 0.1 g/kg xanthan gum (My Protein, Northwich, UK) was added and the mixture was blended thoroughly. This resulted in a viscous mixture similar in consistency to soft-set jelly which was not possible for subjects to simply drink and was required to be consumed from a standard bowl with a standard spoon. During FOOD, subjects consumed a standardised meal consisting of puffed rice cereal, semi-skimmed milk, white bread, seedless strawberry jam, and apple juice. This was selected to provide 20% of estimated energy requirements, determined by multiplying resting metabolic rate [26] by a physical activity level of 1.6, indicating light activity. Subjects ate the cereal and milk in FOOD from a standard bowl using a standard spoon. During WAT, subjects consumed 8 mL/kg body mass of plain tap water. Tap water was consumed alongside the PLA and FOOD meals, to ensure iso-volume total water content of all three meals. The nutritional contents of the breakfast meals are presented in Table 2.

**Table 2:** Nutritional content of the breakfast meals

	WAT		PLA		FOOD	
	Mean	SD	Mean	SD	Mean	SD
Carbohydrate (g)	0	0	1.4	0.1	114.9	5.9
Protein (g)	0	0	0.3	0	15.7	0.8
Fat (g)	0	0	0	0	5.1	0.3
Fibre (g)	0	0	4.8	0.4	2.4	0.1
Energy (kcal)	0	0	16	1	573	30
Energy (kJ)	0	0	68	6	2399	124
Water (mL)	618	54	618	54	618	54
Volume (g)	618	54	625	55	757	60

*WAT* water-only control, *PLA* placebo breakfast, *FOOD* typical whole-food breakfast

### Ad libitum lunch meal

The ad libitum lunch meal consisted of pasta, tomato sauce and extra virgin olive oil (Sainsburys, UK). The meal was standardised across all subjects and trials, and was homogenous in nature, providing 1.25 ± 0.01 kcal/g (69% carbohydrate, 11% protein, 18% fat, and 2% fibre). The meal was prepared in excess of expected consumption and in advance of experimental trials using standardised cooking and cooling procedures and was re-warmed prior to serving. Subjects ate this meal in a custom-built booth to ensure external and social interactions did not interfere with food intake. Directly outside the booth, a table was set up behind a screen to ensure complete privacy. On this table, a serving spoon and a large plastic bowl containing the entire lunch meal were placed and subjects were required to self-serve pasta into a smaller bowl before returning to the booth to eat with the cutlery provided. Subjects were able to repeat this process as many times as they desired within the allotted 20 min but were explicitly instructed to eat until they felt “comfortably full and satisfied”. Ad libitum water intake was permitted during the eating period. Food and water were weighed before and after the eating period to quantify intakes. Subjects were required to remain in the booth for the entire 20-min period, even if they had ceased eating. All subjects had voluntarily ceased eating within the allotted 20 min in all trials.

### Subjective appetite responses

Subjects rated their subjective sensations of hunger, fullness, desire to eat (DTE), prospective food consumption (PFC), and nausea on paper-based 100 mm visual analogue scales (VAS) [27]. Ratings of subjective sensations of alertness, satisfaction, tiredness, relaxation, and energy were included as decoy questions to distract subjects from the main study outcomes. VAS had written anchors of “not at all/no desire at all/none at all” and “extremely/a lot” placed at 0 and 100 mm, respectively.

### Blood sampling and analysis

Venous blood samples (~ 10 mL per sample) were collected via venepuncture of the antecubital vein. The first 2 mL of each sample was discarded and then 2.7 mL of blood was collected into an EDTA tube (1.6 mg/mL; Sarstedt AG & Co, Nümbrecht, Germany) containing a solution (10 µL/mL of blood) of potassium phosphate buffer (PBS) (0.05 M), *P*-hydroxymercuribenzoic acid (PHMB) (0.05 M), and sodium hydroxide solution (NaOH) (0.006 M) for determination of acylated ghrelin concentration by a commercially available ELISA (CV 4.7–8.7%; LoD < 5 pg/mL; Bertin Technologies, Montigny le Bretonneux, France). A further 4.9 mL of blood was collected into an EDTA tube (1.6 mg/mL) for measurement of total PYY concentration using a commercially available ELISA (CV 4.0–4.9%; LoD 5.6 pg/mL; Merck Millipore Ltd, Watford, United Kingdom). Following collection, venous blood samples were centrifuged (1700*g*, 15 min, 4 °C), and the resultant plasma was stored at -80 °C until analysis. Capillary blood samples were collected by piercing the fingertip (Unistick 3 Extra, Owen Mumford, UK). The first drop of blood was discarded and a free-flowing capillary blood sample (20 µL) was collected into a glass capillary tube which was then added to 1 mL of a haemolysing solution. This solution was thoroughly mixed before being analysed immediately using a desktop blood glucose analyser (CV: 3.3%; Biosen, EKF Diagnostics, Cardiff, UK). Due to an issue with venous blood collection, one subject’s venous blood samples were omitted from the final analysis.

### Statistical analyses

Data were analysed using SPSS v26.0 (IBM, Chicago, USA). All data were checked for normality of distribution using a Shapiro–Wilk test. For subjective appetite-related variables and blood glucose concentrations, total area under the curve (AUC) values were calculated using the trapezoidal method. Data containing one factor (baseline measurements, energy/water intake, and AUC values) were analysed using one-way repeated-measures ANOVA. Data containing two factors (appetite sensations, blood glucose, plasma acylated ghrelin, and PYY concentrations) were analysed using repeated-measures ANOVA. Significant ANOVA main effects were explored with post-hoc paired samples *t*-tests for normally distributed data, or Wilcoxon Signed-Rank tests for non-normally distributed data. Holm-Bonferroni stepwise adjustments for multiple comparisons were made to reduce type I error rate. For plasma acylated ghrelin concentrations, box plot analyses showed two consistently outlying subjects within the data set, exhibiting concentrations ~ 5 and ~ 10 SD greater than the mean of the eleven other subjects. Therefore, these subjects were removed from the analysis of acylated ghrelin data. Data sets were determined to be statistically different when *P* < 0.05. Data are presented as mean ± 1SD, unless otherwise stated. Where appropriate and to supplement key findings, effect sizes (ES; Cohen’s *d*_*z*_) were calculated for within-measures comparisons with 0.2, 0.5, and 0.8 representing small, medium, and large ES, respectively [28].

## Results

### Ad libitum food and water intake

**Fig. 1:**
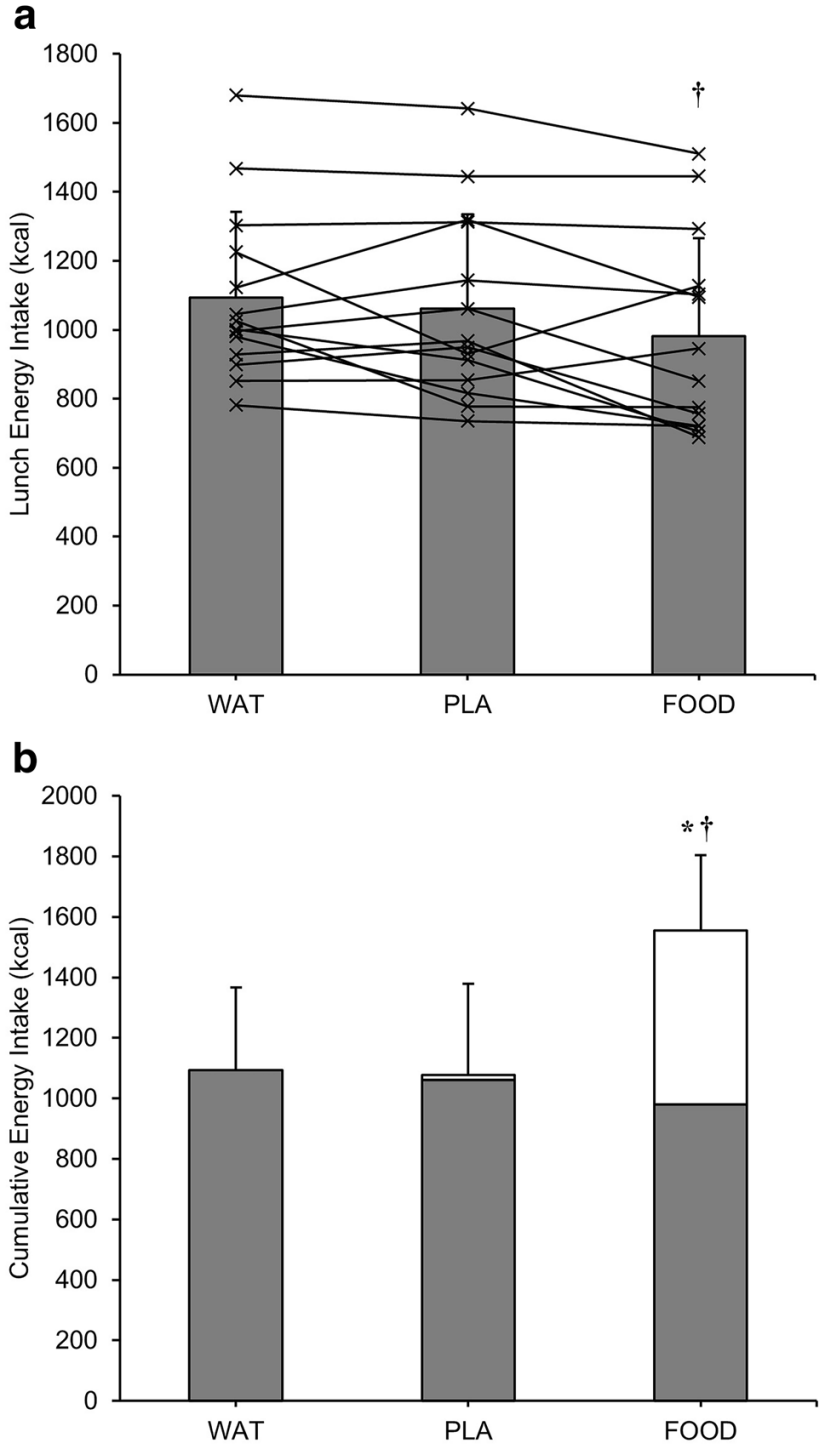
**a** Ad libitum energy intake (kcal) at lunch and **b** cumulative energy intake (kcal) across the entire trial. The bars display mean values at lunch and breakfast, with vertical error bars representing SD. The lines display individual subjects’ lunch energy intake for each experimental trial. ^†^*P* < 0.05 FOOD vs WAT; *P < 0.05 FOOD vs PLA

Ad libitum energy intake at lunch was significantly greater during WAT (1093 ± 249 kcal) compared to FOOD (981 ± 284 kcal; *d*_*z*_ = 0.91; *P* < 0.05), with PLA (1062 ± 273 kcal) not different from WAT (*d*_*z*_ = 0.24; *P* = 1.000) or food (*d*_*z*_ = 0.60; *P* = 0.088) (Fig. 1a). There was no effect of trial order on ad libitum energy intake (*P* = 0.696). Combining energy intake at lunch with the energy contained in each breakfast meal, cumulative energy intake during FOOD (1554 ± 301 kcal) was greater than during PLA (1078 ± 274 kcal; *d*_*z*_ = 3.49; *P* < 0.001) and WAT (1093 ± 249 kcal; *d*_*z*_ = 3.32; *P* < 0.001). Cumulative energy intake was not different between PLA and WAT (*d*_*z*_ = 0.11; *P* = 1.000; Fig. 1b). There were no differences in ad libitum water intake at lunch between trials (PLA: 397 ± 211 mL; FOOD: 373 ± 171 mL; WAT: 376 ± 154 mL; *P* = 0.768).

### Subjective appetite responses

**Fig. 2:**
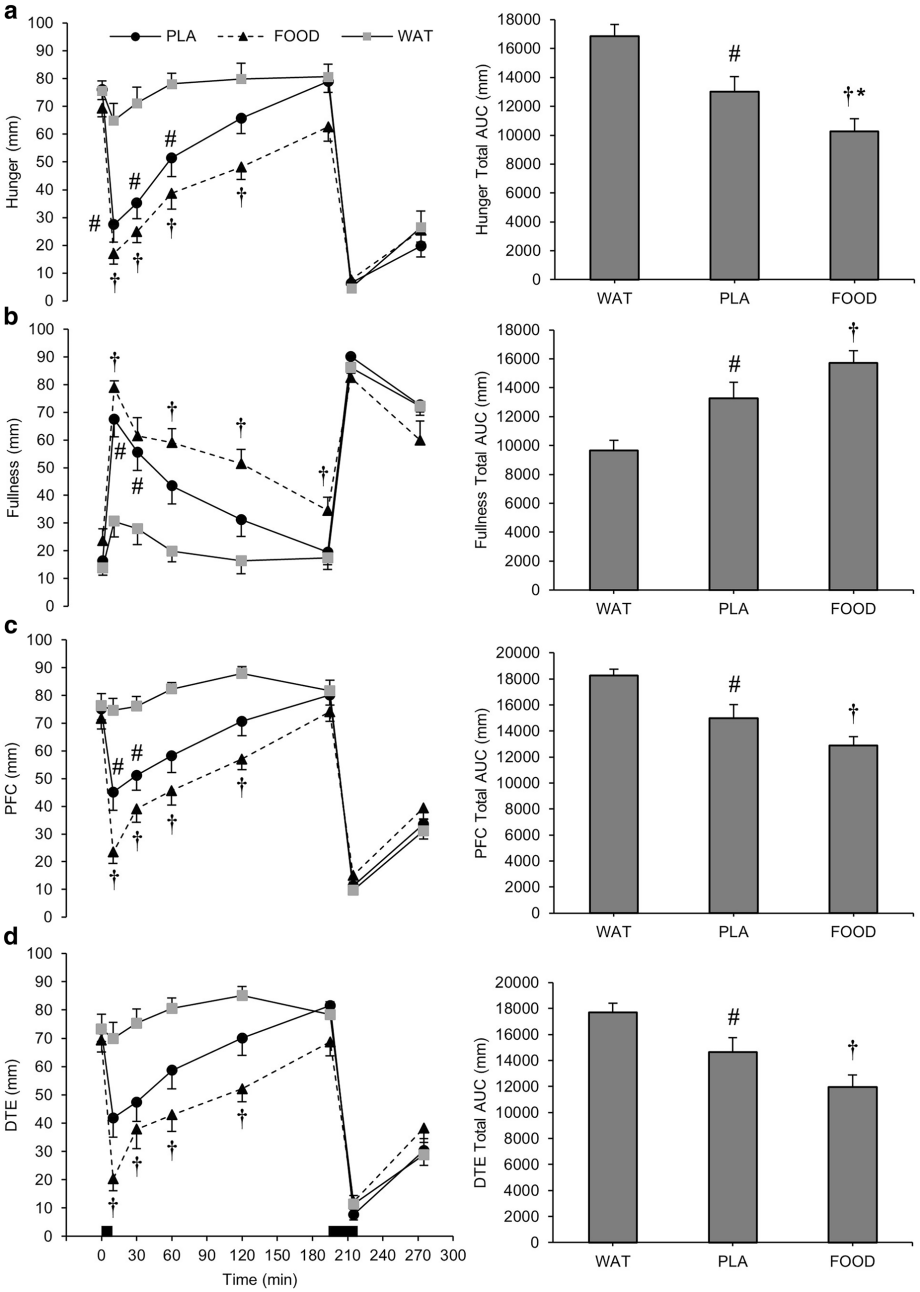
**a** Hunger, **b** fullness, **c** prospective food consumption (PFC), and **d** desire to eat (DTE) during WAT, PLA, and FOOD. Data are presented at each time point (left) and as total area under the curve (AUC) for each trial (right). ^†^*P* < 0.05 FOOD vs WAT; *P < 0.05 FOOD vs PLA; ^#^*P* < 0.05 PLA vs WAT. Black rectangles represent breakfast and lunch. Data are mean ± SEM

**Fig. 3:**
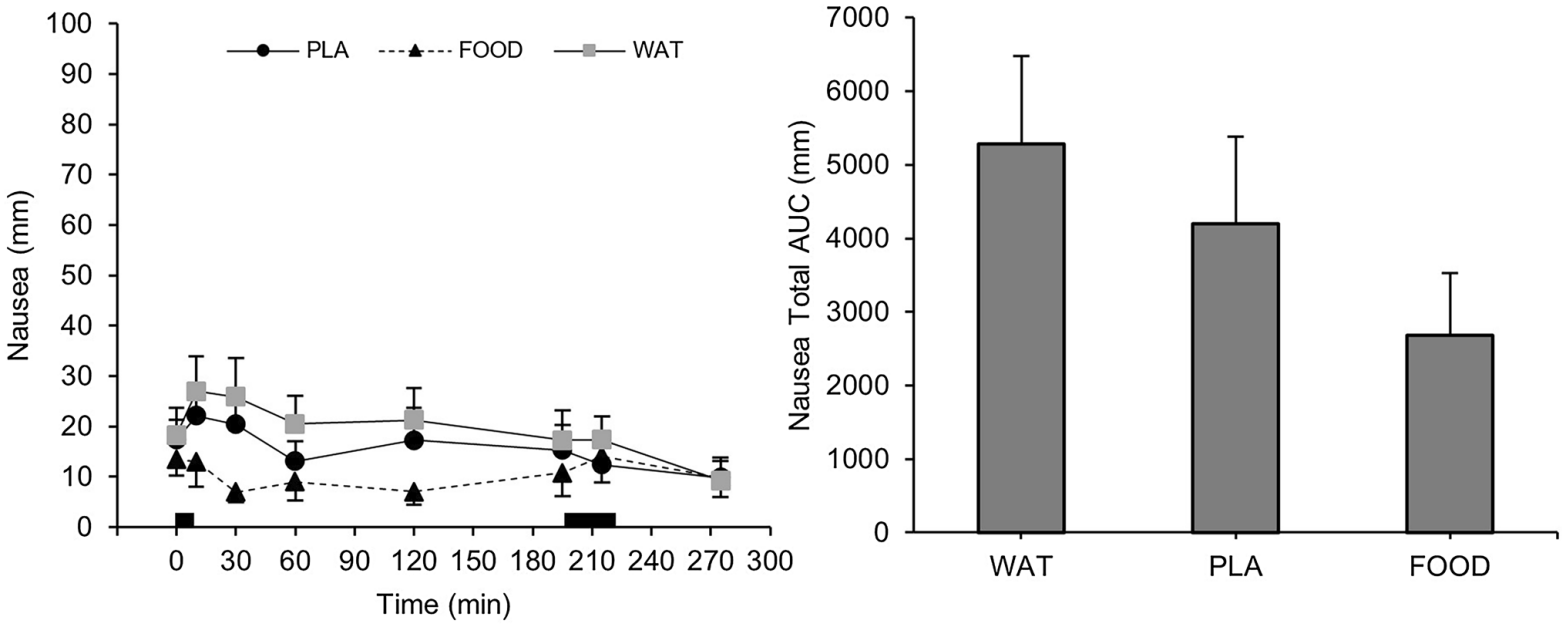
Nausea during WAT, PLA, and FOOD. Data are presented at each time point (left) and as total area under the curve (AUC) for each trial (right). Black rectangles represent breakfast and lunch. Data are mean ± SEM

There were trial (*P* < 0.001), time (*P* < 0.001), and interaction (*P* < 0.001) effects for hunger, fullness, PFC, and DTE. There were no significant effects for nausea (*P* ≥ 0.081). AUC for hunger (*d*_*z*_ = 0.79), DTE (*d*_*z*_ = 0.69), and PFC (*d*_*z*_ = 0.80) were lower, and fullness was higher (*d*_*z*_ = 0.71), during PLA compared to WAT (*P* < 0.05). AUC for hunger (*d*_*z*_ = 1.63), DTE (*d*_*z*_ = 1.43), and PFC (*d*_*z*_ = 2.05) were also lower, and fullness was also higher (*d*_*z*_ = 1.38), during FOOD compared to WAT (*P* < 0.001; Fig. 2). Additionally, AUC for hunger was lower during FOOD compared to PLA (*d*_*z*_ = 0.60; *P* < 0.05). AUC for nausea was not different between trials (*P* = 0.070; Fig. 3).

Following breakfast consumption, hunger was lower during PLA and FOOD, compared to WAT, for 60 min (*P* < 0.05) and remained lower in FOOD for 120 min (*P* < 0.05). Hunger was not different between trials immediately before lunch (*P* ≥ 0.091). Fullness was higher in PLA compared to WAT at 10-min and 30-min post-breakfast (*P* < 0.05). Fullness was significantly greater in FOOD compared to WAT at all time points until immediately before lunch (*P* < 0.05), except for 30 min (*P* = 0.064). PFC was lower in PLA and FOOD compared to WAT for 30-min post-breakfast (*P* < 0.05) and remained lower in FOOD until 120 min (*P* < 0.01). DTE was lower in FOOD, compared to WAT, for 120 min after breakfast (*P* < 0.01), but there were no differences between PLA and FOOD (*P* ≥ 0.126) or PLA and WAT (*P* ≥ 0.066) at any time point.

### Blood analyses

**Fig. 4:**
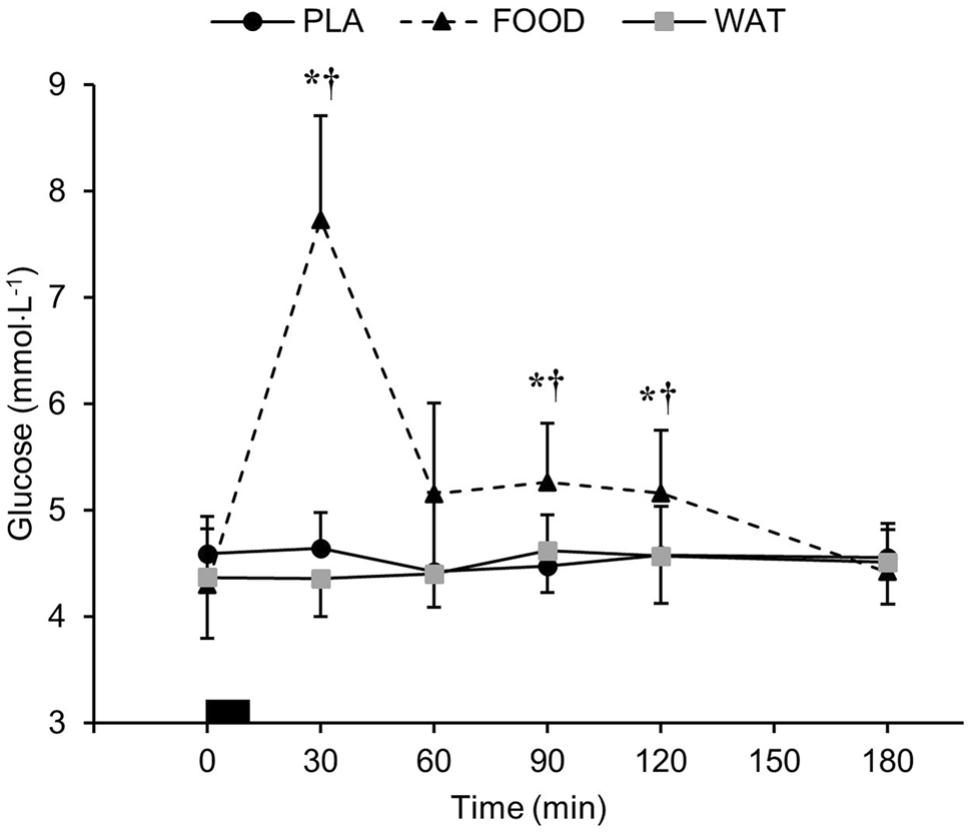
Blood glucose concentrations over the course of the trial during WAT, PLA, and FOOD. ^†^*P* < 0.05 FOOD vs WAT; *P < 0.05 FOOD vs PLA. Data are mean ± SD

There were time (*P* < 0.001), trial (*P* < 0.001), and interaction (*P* < 0.001) effects for blood glucose concentrations. Compared to FOOD, blood glucose concentrations were lower during PLA and WAT at 30 (*P* < 0.001), 90 (*P* < 0.01), and 120 min (*P* < 0.05). Blood glucose concentrations increased after breakfast during FOOD and were significantly greater than baseline between 30 and 120 min (*P* < 0.01), returning to baseline concentrations at 180 min (*P* = 0.501). Blood glucose concentrations did not change from baseline during PLA (*P* ≥ 0.883) or WAT (*P* ≥ 0.302; Fig. 4). AUC for blood glucose concentrations was significantly different between trials (*P* < 0.001). AUC was significantly higher in FOOD compared to PLA (*P* < 0.001) and WAT (*P* < 0.001). There was no difference in AUC for glucose between PLA and WAT (*P* = 0.482).

**Fig. 5:**
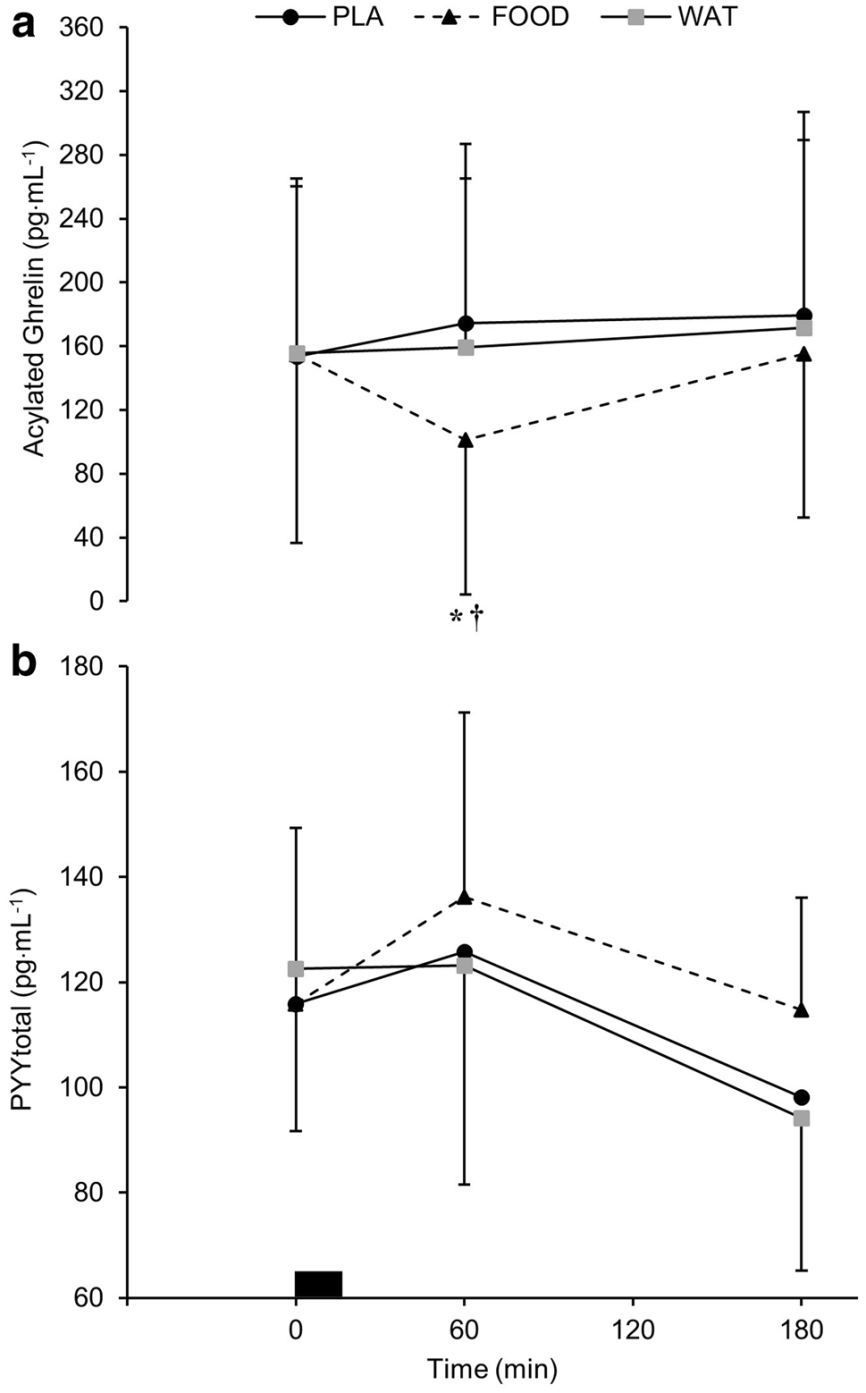
**a** Plasma acylated ghrelin (*n* = 11) and **b** plasma PYY_total_ (*n* = 13) concentrations over the course of the trial during WAT, PLA, and FOOD. ^†^*P* < 0.05 FOOD vs WAT; *P < 0.05 FOOD vs PLA. Black rectangle represents breakfast. Data are mean ± SD

There were time (*P* < 0.001), trial (*P* < 0.001), and interaction (*P* < 0.001) effects for plasma acylated ghrelin concentrations. Acylated ghrelin concentrations were lower at 60 min during FOOD compared to PLA and WAT (*P* < 0.05). Acylated ghrelin concentrations were greater than baseline at 60 and 180 min in PLA (*P* < 0.01), and at 180 min during WAT (*P* < 0.05). Acylated ghrelin concentrations were lower than baseline at 60 min in FOOD (*P* < 0.01; Fig. 5a).

Plasma PYY concentrations showed a main effect of time (*P* < 0.001), but there were no main effects of trial (*P* = 0.187), and no interaction effects (*P* = 0.054; Fig. 5b).

## Discussion

The aim of this study was to examine appetite responses and energy intake at an ad libitum lunch following consumption of a very-low-energy, viscous placebo breakfast meal, compared with a typical whole-food breakfast and a water-only control. Subjective appetite was suppressed during PLA and FOOD compared to WAT, although energy intake at lunch was lower only during FOOD, but not PLA, compared to WAT. Nevertheless, due to the very low energy content of the placebo breakfast meal, cumulative energy intake (breakfast plus lunch) across the PLA trial period was lower than FOOD, and not different to WAT. These results support the idea that breakfast omission may successfully reduce energy intake over breakfast and lunch. Furthermore, consumption of a very-low-energy, viscous placebo breakfast may attenuate the elevations in subjective appetite associated with breakfast omission, potentially enhancing its efficacy by reducing the likelihood of mid-morning snacking and improving dietary adherence.

With the exception of one study which provided a notably small breakfast (~ 250 kcal) [15], breakfast omission studies show that the energy deficit created by omitting breakfast is not fully compensated for at lunch, and, as such, cumulative energy intake is reduced compared to when breakfast is consumed [9, 10, 12, 16, 29]. This was also the case in this study, as, compared to FOOD, cumulative energy intake was approximately 477 and 461 kcal lower during PLA and WAT. Whilst it is possible that further energy intake compensation may occur at subsequent meals, studies that have examined energy intake beyond a single meal have revealed no such compensation [10, 16]. Collectively, these studies suggest that the effects of breakfast omission on ad libitum energy intake are largely constrained to lunch. These findings support the hypothesis that total daily energy intake can be similarly reduced following both complete breakfast omission and breakfast omission instigated via the consumption of a very-low-energy placebo breakfast.

We have shown a 112 kcal increase in lunch energy intake when a breakfast containing ~ 575 kcal was omitted, compared to consumed. This is consistent with previous studies, which have reported an increase in lunch energy intake of between 153 and 206 kcal following breakfast omission, compared to when a breakfast containing ~ 250–733 kcal was consumed [10, 12, 15, 16]. Some studies, however, have reported a similar energy intake at lunch following breakfast omission and consumption [9, 16, 29]. Inconsistencies in these findings may result from methodological differences between studies, such as differences in the time interval between breakfast and lunch, and/or the method employed to assess ad libitum energy intake (i.e. a homogenous, single-item meal versus a multi-item buffet meal).

Acute, single-exposure studies support the efficacy of breakfast omission for the reduction of energy intake over the course of a day. Findings from longer-term studies, however, suggest that some degree of adaptation may occur when breakfast is omitted over consecutive days. In a cross-over study, two weeks of daily breakfast omission resulted in greater self-reported daily energy intake in a sample of healthy, lean females [17]. Additionally, individuals with obesity either omitted, or consumed, a 700-kcal breakfast (before 11:00) daily for six weeks and it was found that breakfast omission led to a compensatory increase in energy intake after 11:00, which ultimately resulted in no difference in total daily energy intake between the groups [18]. These data suggest that breakfast omission over the longer term may be associated with adaptations that drive an increase in appetite and energy intake to account for the energy omitted at breakfast.

Naharudin et al. [20] reported that a placebo breakfast meal suppressed appetite compared to water only. In line with this, the current study showed that appetite was supressed during both PLA and FOOD compared to WAT. Specifically, hunger, PFC and DTE were lower, and fullness was higher during the PLA and FOOD trials, compared to WAT. The regulation of appetite is important as dietary success is known to be influenced by persistently elevated appetite sensations [5]. Dietary self-control, or ‘willpower’, appears to be negatively associated with increased levels of hunger, for example, hungry individuals typically exhibit poorer food choices by selecting more high-calorie or ‘junk food’ options [30, 31]. Furthermore, increased hunger led to individuals underestimating their self-belief in achieving dietary success, which worsened their dieting intentions [32]. In this study, the suppression of appetite during PLA was most pronounced 30–60 min after breakfast, whereas FOOD suppressed appetite for longer. This indicates that consuming a placebo breakfast does not suppress appetite as strongly as after consuming a ~ 575-kcal whole-food breakfast. However, the transient suppression of appetite during PLA may be meaningful, as research has also linked breakfast omission with increased impulsive snacking [33]. Therefore, the immediate appetite suppressing effects of consuming a very-low-energy placebo breakfast that occur between breakfast and lunch have the potential to improve dietary success by increasing restraint and reducing the temptation for snacking during the mid-morning. Future research should aim to elucidate the effects of placebo breakfast consumption on dietary adherence and snacking behaviours in a free-living environment.

The viscosity of the PLA breakfast was increased by the addition of xanthan gum, a soluble fibre often used as a low-energy thickening agent [34]. The effects of several different viscous soluble fibres, including pectin, alginate, and β-glucan, on appetite and energy intake, have been examined in a number of studies with differing methodological designs [25, 35]. Typically, these studies compare the satiating properties of soluble fibre mixtures of varying viscosities [36, 37], and/or nutritional contents [38, 39]. It is generally agreed that increasing the viscosity of a liquid enhances its effects on satiety [36, 38–40] and food intake [35, 37]. The study of Marciani and Colleagues [38] compared the appetite responses to test breakfast meals of both a high and low viscosity, which either contained ~ 323 kcal, or contained no energy. The meals of increased viscosity resulted in greater subjective satiety ratings, independent of the presence or absence of energy. Similar findings were observed by Solah et al. [39], who found that the viscosity of a test beverage had a greater effect on satiety than its protein content. These results suggest that the addition of soluble fibre to a meal may have more profound effects on appetite than its nutrient content. We extend these findings by comparing both the appetite and energy intake responses to a very-low-energy, viscous meal with those of an ecologically valid, whole-food meal with an energy content in line with what may be consumed at breakfast in the real-world.

It is interesting to note that despite having a virtually identical energy and macronutrient content, PLA and WAT produced divergent appetite responses during the early post-breakfast period. The PLA breakfast contained a small amount of energy (16 ± 1 kcal), although data from our physiological variables indicated that this is unlikely to explain the differences in appetite between PLA and WAT. Acylated ghrelin and PYY are orexigenic and anorexigenic hormones, respectively [13, 14], and respond predominantly to the ingestion of energy, rather than gastric distension [41, 42]. Accordingly, the only changes observed in these physiological markers of appetite and blood glucose concentrations were after consumption of the energy-containing breakfast during the FOOD trial. Aligning with this, plasma concentrations of acylated ghrelin and PYY were not different between the WAT and PLA trials. Therefore, despite the FOOD breakfast inducing a hormonal response associated with increased satiety and reduced hunger, these physiological variables cannot explain differences in subjective appetite between the PLA and WAT trials. It should be noted that the physiological regulation of appetite is complex, and the effects of other hormones and/or neural signals on appetite during PLA cannot be ruled out.

Such discordant hormonal and subjective appetite responses have been observed previously following placebo breakfast consumption [20]. Subjects in this study and that of Naharudin et al*.* [20] were self-reported regular breakfast consumers, and research suggests that breakfast omission adversely affects appetite to a greater extent in habitual breakfast consumers than breakfast skippers [43]. Therefore, simply the knowledge of having consumed breakfast, rather than the physiological responses to ingested nutrients, may mediate the satiating effects of breakfast consumption in these individuals. Additionally, consuming a volume of water immediately prior to a meal has been shown to reduce appetite and ad libitum energy intake, likely via gastric distension [44], although the gastric emptying rate of water is rapid [45], and its effects on appetite are typically lost after 30 min in young individuals [46]. Gastric emptying is, however, slowed in semi-solid meals by the addition of soluble fibre [47]. Because the addition of xanthan gum to PLA provided a small amount of fibre (~ 5 g), a delayed gastric emptying of PLA compared to WAT is a possible mechanism explaining the divergent appetite responses to the meals. Additionally, the oral processing of food which includes chewing and swallowing mediates the satiating effects of a meal via physiological and psychological mechanisms [48]. As such, the prolonged oro-sensory exposure time of more solid foods has been shown to elicit a greater and extended suppression of subjective appetite, compared to liquid foods [49]. This may also contribute to the differences in subjective appetite between PLA and WAT.

Long-term weight management is dependent upon the interplay between energy intake and energy expenditure [4], and it has been previously reported that 6 weeks of breakfast omission resulted in a reduction in habitual physical activity energy expenditure which fully compensated for the reduction in energy intake, thus offsetting the energy deficit created by the omission of breakfast [50]. Whether a similar effect would be shown when a very-low-energy placebo breakfast is consumed, rather than skipping the breakfast meal entirely, is unknown. It is interesting to note, however, that two previous studies showed that endurance and resistance exercise performance was greater after consumption of a very-low-energy placebo breakfast, compared to water only [20, 51]. Therefore, it is plausible that the act of eating (rather than the specific content of the meal) in the morning is sufficient to maintain physical activity, and as such, this may present a more effective method of energy restriction. This warrants further investigation.

Herein, we provide novel data demonstrating that an acute, single-exposure to placebo breakfast consumption can suppress subjective appetite compared to consuming water only, and can reduce energy intake over breakfast and lunch, compared to a typical breakfast meal. These findings have practical implications for lean individuals looking to manage energy intake as a means of weight maintenance. Future studies should explore whether similar results would be observed following multiple exposures to placebo breakfast consumption over days and weeks, especially given the initial unfamiliarity of the viscous breakfast to subjects. Furthermore, to increase the application of this intervention, it would be prudent to examine responses to a placebo breakfast within an unblinded study design to account for potential demand effects resulting from knowledge of its lack of energy content. Finally, the effects of placebo breakfast consumption should be investigated in other population groups, specifically overweight or obese individuals, who have been shown to respond differently to acute and chronic breakfast omission [9, 12, 18, 50].

In conclusion, a typical, whole-food breakfast and a very-low-energy placebo breakfast both reduced subjective appetite compared to water, but the placebo breakfast also reduced cumulative energy intake across breakfast and lunch. Therefore, placebo breakfast consumption may be an effective strategy for managing the elevations in appetite which often accompany breakfast omission, whilst still reducing cumulative energy intake over breakfast and lunch, and thus aiding weight management.

## Supplementary Information

Below is the link to the electronic supplementary material.

Supplementary file1 Raw dataset for the experimental study is available as online supporting information (XLSX 109 KB)

## Data Availability

The data described in this article will be available as supplementary electronic material.
